# Explosion of Dust

**Published:** 1881-10

**Authors:** 


### EXPLOSION OF DUST.

The explosion of malt dust, causing a fire, throwing down the walls and blowing off the roof of Mr. Geo. Ehret's beer brewery in this city, one of the finest in the country, should be a warning to proprietors and employees of all factories and buildings where dust is mixed with the air. Dust formed from any combustible material when a sufficient quantity is suspended in the air is an explosive, and in some cases very violent and destructive, as was the malt dust in Mr. Ehret's brewery and many other similar disasters.

